# Vitamin D Status, Bone Mineral Density, and *VDR* Gene Polymorphism in a Cohort of Belarusian Postmenopausal Women

**DOI:** 10.3390/nu13030837

**Published:** 2021-03-04

**Authors:** Pavel Marozik, Alena Rudenka, Katsiaryna Kobets, Ema Rudenka

**Affiliations:** 1 Laboratory of Human Genetics, Institute of Genetics and Cytology of the National Academy of Sciences of Belarus, 220072 Minsk, Belarus; E.Kobets@igc.by; 2 Department of General Biology and Genetics, International Sakharov Environmental Institute of the Belarusian State University, 220070 Minsk, Belarus; 3 Department of Cardiology and Rheumatology, Belarusian Medical Academy of Post-Graduate Education, 220013 Minsk, Belarus; alenka.v.ru@gmail.com; 4 Department of Cardiology and Internal Diseases, Belarusian State Medical University, 220116 Minsk, Belarus; rudenka.ema@gmail.com

**Keywords:** vitamin D, *VDR* gene, polymorphism, predisposition, osteoporosis, bone mineral density

## Abstract

Vitamin D plays an important role in bone metabolism and is important for the prevention of multifactorial pathologies, including osteoporosis (OP). The biological action of vitamin is realized through its receptor, which is coded by the *VDR* gene. *VDR* gene polymorphism can influence individual predisposition to OP and response to vitamin D supplementation. The aim of this work was to reveal the effects of *VDR* gene ApaI rs7975232, BsmI rs1544410, TaqI rs731236, FokI rs2228570, and Cdx2 rs11568820 variants on bone mineral density (BMD), 25-hydroxyvitamin D level, and OP risk in Belarusian women. Methods. The case group included 355 women with postmenopausal OP, and the control group comprised 247 women who met the inclusion criteria. TaqMan genotyping assay was used to determine *VDR* gene variants. Results. Rs7975232 A/A, rs1544410 T/T, and rs731236 G/G single variants and their A-T-G haplotype showed a significant association with increased OP risk (for A-T-G, OR = 1.8, *p* = 0.0001) and decreased BMD (A-T-G, −0.09 g/cm^2^, *p* = 0.0001). The rs11568820 A-allele showed a protective effect on BMD (+0.22 g/cm^2^, *p* = 0.027). A significant dose effect with 25(OH)D was found for rs1544410, rs731236, and rs11568820 genotypes. Rs731236 A/A was associated with the 25(OH)D deficiency state. Conclusion. Our novel data on the relationship between *VDR* gene variants and BMD, 25(OH)D level, and OP risk highlights the importance of genetic markers for personalized medicine strategy.

## 1. Introduction

Osteoporosis (OP) is defined as a systemic skeletal disease characterized by low bone mineral density (BMD) and a microarchitectural deterioration of bone tissue, leading to increased bone fragility and susceptibility to fracture [1]. Postmenopausal osteoporosis (PMO) is the most common form of primary OP, affecting menopausal women. The clinical significance of OP lies in its serious complications such as low-energy fractures, causing an increased risk of morbidity and mortality, especially in the elderly [2]. The impact of OP and fragility fractures on human health is huge: more than 9 million osteoporotic fractures are registered annually in the world [2]. Such fractures are associated with 26,300 life years lost and 1.16 million quality-adjusted life years (QALYs) lost yearly in EU countries, and the costs of treatment of osteoporotic fractures in 2010 has been estimated at €37 billion [3]. Projected demographic changes will cause an increase in fracture burden in coming decades [4].

The pathogenesis of OP is complex and includes many factors, among which genetic factors are of particular importance, as up to 90% of susceptibility to OP may be genetically determined [5]. However, the influence of environmental factors on the risk of OP should not be underestimated. In accordance with modern concepts, vitamin D is a steroid prohormone, which, along with parathyroid hormone (PTH) and calcitonin, plays a major role in the regulation of genes involved in calcium–phosphorus and bone metabolism [6]. Vitamin D effects are mediated by its binding to a specific steroid receptor (vitamin D receptor, VDR) that has a transcription factor activity [7]. The formation of the vitamin D steroid receptor complex results in the activation or silencing of numerous target genes, regulating bone remodeling, calcium homeostasis, and immune response.

The human *VDR* gene is located on the 12th chromosome (12q12-14) and consists of 14 exons spanning about 75 kb: eight protein-coding exons (2–9), six untranslated exons (1A–1F), located on the non-coding 5′region, and several promoter regions that are DNA sequences recognized by RNA polymerase as a launching pad for the initiation of specific transcription [8]. Even a small modification in a gene may affect the structure and functional activity of the receptor. Common single nucleotide variations (SNVs) in the *VDR* gene may be associated with different biological responses to vitamin D. The *VDR* ApaI (rs7975232, c.1025-49C>A), BsmI (rs1544410, 1024+443C>T), and TaqI (rs731236, c.1056A>G) SNVs are located at the 3′-untranslated end. They do not alter the amino acid sequence of the encoded protein but influence gene expression, regulating mRNA stability [9]. The *VDR* FokI variant (rs2228570, c.2T(A, f)>C(G, F), p.Met1Arg) is located in the coding region of the *VDR* gene (exon 2) and leads to the loss of the ATG translation initiation region, resulting in a shorter and more active receptor protein [10]. *VDR* Cdx2 G-to-A (rs11568820) substitution is located in the promoter region and causes 30% increased transcriptional activity [11].

Although several studies on different populations revealed an association of *VDR* gene variants with BMD [12,13] and serum 25(OH)D [14,15], many issues in this area are not fully understood. An investigation of *VDR* gene polymorphisms may help clarify criteria for the identification of individuals with high risk of PMO and thus conduct a timely set of preventive measures in target risk groups as well as evaluate effectiveness of therapy [16].

The aim of our study was to investigate the relationship between *VDR* gene single variants and haplotypes and PMO risk, BMD, and serum 25(OH)D level in Belarusian postmenopausal women.

## 2. Materials and Methods

### 2.1. Study Subjects

This study was a cross-sectional cohort study conducted at the outpatient department and inpatient clinic. Patients were recruited at the Minsk City Center for Osteoporosis and Bone-Muscular Diseases Prevention and Rheumatologic Department of 1st Minsk city clinic (Minsk, Belarus). The study protocol was approved by the Local Research Ethics Committee of Belarusian Medical Academy of Postgraduate Education. White Caucasian women were screened for participation. Inclusion criteria were wiliness to participate in the study, female sex, duration of menopause at least 3 years, and established diagnosis of OP according to World Health Organization Diagnostic Criteria [17]. Exclusion criteria: presence of other metabolic bone diseases (such as Paget’s disease and osteomalacia), diseases, affecting bone metabolism (such as endocrine osteopathy, renal failure, Cron’s disease, rheumatic diseases etc.), malignant tumors, use of medications likely influencing BMD. After assessing compliance with inclusion and exclusion criteria, all the enrolled women signed written informed consent for participation in the study in accordance with the declaration of Helsinki (as revised in 2013). Participants of the study have filled out questionnaires to identify clinical risk factors for OP (age of menopause, history of fractures etc.).

### 2.2. Clinical Evaluation

BMD was evaluated by DXA (GE Lunar, Madison, WI, USA). Calibration of the device was performed daily using a standard spine phantom provided by the manufacturer. Lumbar spine (LS, L1–L4) and femoral neck (FN) BMD (g/cm^2^) was measured on the same machine. Diagnosis of OP was established on the basis of T-score criteria for Caucasian women [17].

Fasting blood samples for biochemical and electrochemiluminescence blood tests were obtained from the cubital vein in the morning, not earlier than 10–12 h after the last meal, into a sterile vacuum Vacutainer tube without additives. Determination of serum vitamin D was performed by electrochemiluminescence immunoassay on the Cobas e411 analyzer (Roche Diagnostic, Rotkreuz, Switzerland). All patients were concealed about adequate calcium dietary intake and were taking calcium supplementation in the form of calcium carbonate equivalent to 500 mg elemental calcium. All subjects were supplemented with a daily dose of 400–800 international units (IU) of cholecalciferol according to European guidance [18]. In accordance with international recommendations, the level of vitamin D was considered appropriate at 25(OH)D value > 30 ng/mL, insufficiency was diagnosed at rates of 20‒30 ng/mL, and 25(OH)D concentration less than 20 ng/mL was considered as vitamin D deficiency [19].

### 2.3. Genotyping

Genomic DNA was isolated from whole blood using the standard phenol–chloroform extraction, the concentration and purity were measured using a NanoDrop 8000 spectrophotometer (Thermo Fisher Scientific, Wilmington, NC, USA). Information on *VDR* gene variants was obtained from the Entrez Gene database (www.ncbi.nlm.nih.gov/gene, accessed on 5 June 2020). Selected SNVs (ApaI rs7975232, BsmI rs1544410, TaqI rs731236, FokI rs2228570 and Cdx2 rs11568820) were determined using the quantitative polymerase chain reaction (PCR) with TaqMan Probes (Thermo Fisher Scientific, Waltham, MA, USA) in the CFX96™ Touch Real-Time PCR Detection Systems (Bio-Rad Laboratories, Hercules, NY, USA) as previously described [20,21]. The whole reacting volume in PCR tubes was 10 µL, including 5 µL iTaq™ Universal Probes Supermix (Bio-Rad Laboratories, Hercules, NY, USA), 3.75 µL of mQ water, 0.25 µL × 40 TaqMan™ SNP Genotyping Assay, and 1 µL of genomic DNA (15 ng). The reactions were performed with an initial denaturation at 95 °C for 10 min, followed by 40 cycles of denaturation at 95 °C for 15 s, annealing, and synthesis at 60 °C for 30 s. The final extension was performed at 72 °C for 1 min. Negative and positive controls were randomly included across each PCR run, and several samples were randomly re-genotyped for quality control purposes.

### 2.4. Statistical Analysis

Data analysis was performed using the programming language R. Continuous variables presented as median (25%, 75% interquartile range) and compared using Mann–Whitney U-test. The deviation from Hardy–Weinberg equilibrium was assessed by the chi-square (χ^2^) test. The genetic risk of pathology was estimated using odds ratios, with 95% confidence intervals (CI) and calculated in comparison to reference (major homozygous) genotype. The codominant model was defined and tested for all SNVs. Logistic regression models were used to assess the difference between the characteristics of analyzed groups for categorical data and for comparison of genotype frequencies between these groups. A Multivariate Linear Regression model was used to adjust for confounding factors, and an ANOVA test was used for analysis of continuous variables distribution between genotypes. Beta (β) measures the difference in quantitative traits between genotypes. Pairwise linkage disequilibrium (LD) and haplotype analysis were performed using the R-packages “haplo.stats” (v.1.7.9) and “SNPassoc” (v.1.9-2); the programs used likelihood ratio tests in a generalized linear model and the expectation-maximization algorithm. The differences between the groups were considered statistically significant at *p* < 0.05. *p*-values corrected for multiple testing using the False Discovery Rate (*p* FDR) with Benjamini and Hochberg procedure (n = 5, multiple comparisons).

## 3. Results

In total, 927 subjects were screened for participation in the study, 325 of them were excluded due to eligibility, and 602 of them met inclusion criteria and were allocated to patients with PMO (355 women) and control (247 women) groups, followed by clinical examinations and genetic testing.

### 3.1. Study Subjects Charactetistic

The clinical characteristics of the analyzed cohort are summarized in Table 1. The mean age of all individuals was 62.4 years, the mean weight, height, and BMI were 72.4 kg, 159.8 cm, and 28.4 kg/m^2^, respectively. All participants were ethnic Belarusians.

Cases and controls did not differ in age, age at menopause, and height (Mann‒Whitney *U*-test did not reveal difference, *p* > 0.1). A strong difference between groups was revealed for weight, BMI, lumbar spine, and femoral neck BMD, T-, and Z-scores. The weight and BMI variables were considered potential confounding factors and were adjusted in analysis of association between groups. In the study cohort, 49 patients with PMO had a fracture history (at least one) compared to seven individuals from the control group. The baseline serum 25(OH)D level ranged from 7.4 to 70 ng/mL, and the mean level in all individuals was 25.5 ng/mL. There was a statistically significant difference between analyzed groups in the plasma 25(OH)D level.

### 3.2. The Relationship between VDR Gene Variants and PMO Risk

All subjects were genotyped in the study; the genotype frequencies distribution is presented in Table 2. The five most common polymorphic loci of the *VDR* gene were selected from key publications and studied as candidate markers of PMO. These SNVs with previously established involvement in vitamin D and bone tissue metabolisms were included to the study to validate their effect by analysis of combinations of genetic variants on independent cohorts.

The minor allele frequencies of all analyzed SNVs were not significantly different from those taken from GnomAD data for Europeans [22]. By the analysis, the genotyping data were found to be in correspondence to the expected Hardy–Weinberg equilibrium at the 5% level in the control group (*p* > 0.1). In case group, the deviation from the Hardy–Weinberg equilibrium was revealed only for the rs7975232 variant (*p* = 0.02), which was possibly due to the very low frequency of the T/T homozygous genotype.

The analysis of genotype frequencies of *VDR* gene variants, presented in Table 2, showed significant differences in their distribution between both groups. The most frequent homozygous genotype was taken for reference. Comparing the genotype frequencies between PMO and CON groups, statistically significant differences after FDR correction for multiple testing were found for rs7975232, rs1544410, and rs731236 variants of the *VDR* gene. The PMO group individuals were more likely to carry the rs7975232 A/A genotype (30.4%) compared to the CON group (20.6%, OR = 1.9, 95% CI 1.2‒3.1, *p* FDR = 0.0175). The rs1544410 T/T genotype was significantly over-represented in PMO patients (27.9%) compared to control group (17.0%, OR = 2.4, 95% CI 1.5‒3.8, *p* FDR = 0.0028). For the bearers of the rs731236 G/G homozygous genotype, the risk of osteoporosis was increased (OR = 2.6, 95% CI 1.6‒4.1, *p* FDR = 0.0015). An increased risk of PMO was also revealed for the bearers of the heterozygous genotype A/G, OR = 1.6 (95% CI 1.1‒2.3).

To reduce the potential impact of confounding factors, the association analysis was further adjusted for BMI. When adjusted, the associated gene variants remained significant; additionally, a statistically significant association was revealed for the rs1544410 heterozygous C/T genotype (Table 2).

There was no association with osteoporosis risk found for rs2228570 and rs11568820 variants. Since the rs11568820 T-allele frequency was very low, we used a dominant model of inheritance and merged C/T+T/T genotypes. Despite the absence of significant association after the Yates correction, it can be noted that the frequency of the rs11568820 T/T-genotype is significantly higher in the CON group (4.0%) compared to PMO patients (0.9%).

**Figure 1:**
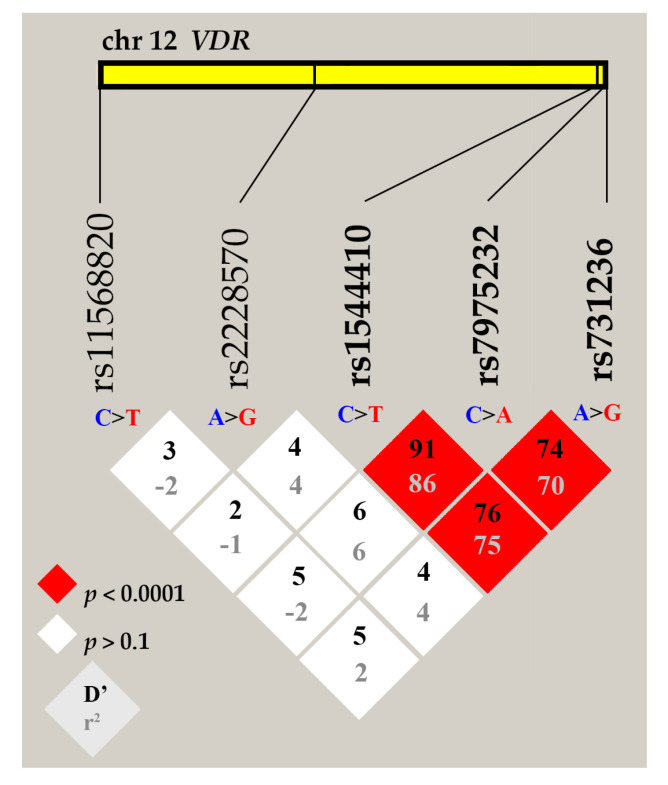
Linkage disequilibrium (LD) plot for rs7975232, rs1544410, rs731236, rs2228570, and rs11568820 variants of the *VDR* gene. LD is displayed as pairwise D’ values multiplied by 100 and given for each SNV combination within each cell. Red cells correspond to a very strong LD; rs7975232, rs1544410, and rs731236 variants are in the same LD block.

Next, we analyzed the pairwise linkage disequilibrium between *VDR* gene variants. An LD plot was constructed using combined genotype data from both groups of individuals (Figure 1).

Performed LD analysis identified one haplotype block, which was composed of *VDR* rs7975232, rs1544410, and rs731236 variants. The measure of linkage strength D’ between rs7975232 and rs1544410 was 91, *p* < 0.0001. The positive coefficient of correlation r^2^ suggests that major alleles of rs7975232, rs1544410, and rs731236 variants are likely to be inherited together, as well as minor alleles. The rs2228570 and rs11568820 variants do not exhibit significant LD, the D’ coefficient ranged for them from 2 to 6; therefore, they were removed from further analysis.

Based on LD data, in further analysis, we combined three *VDR* gene variants from the same block and performed the haplotype analysis. Haplotypes were constructed from all possible allelic combinations and compared between the PMO and control (CON) groups (Table 3).

The haplotype analysis revealed five combinations (C-C-A, A-T-G, A-T-A, C-C-G, and A-C-A) of the possible eight with a frequency greater than 3%. These five haplotypes jointly presented in 96.5% of study subjects. Statistically significant differences between analyzed groups were revealed in the global distribution of allelic combinations (global *p* = 0.00023), suggesting an association of analyzed haplotypes with the risk of PMO. A statistically significant difference was revealed in the distribution of the most frequent C-C-A and A-T-G haplotypes between the PMO and CON groups even after FDR correction for multiple testing. The C-C-A haplotype, constructed from three wild-type alleles, was the most frequent (total frequency 43.4%). This haplotype frequency was significantly higher among controls (49.6%) than among cases (38.6%, *p* FDR = 0.001). The negative haplotype score value of −3.53 suggests that this combination is associated with a decreased risk of PMO. The total frequency of the A-T-G haplotype was 39.4%; it was significantly under-represented in the CON group (31.7%) compared to the PMO group (44.9%, *p* FDR = 0.00005), suggesting that this allelic combination might confer a greater susceptibility to PMO (the highest haplotype score of 4.41 points). In comparison with the most frequent reference (wild-type) haplotype C-C-A, for the bearers of the A-T-G haplotype, the risk of PMO was significantly higher (OR = 1.8, 95% CI 1.4‒2.3, *p* = 0.0001). No significant association was found for other constructed haplotypes.

### 3.3. The Relationship between VDR Gene Variants and Lumbar Spine BMD Level

**Figure 2:**
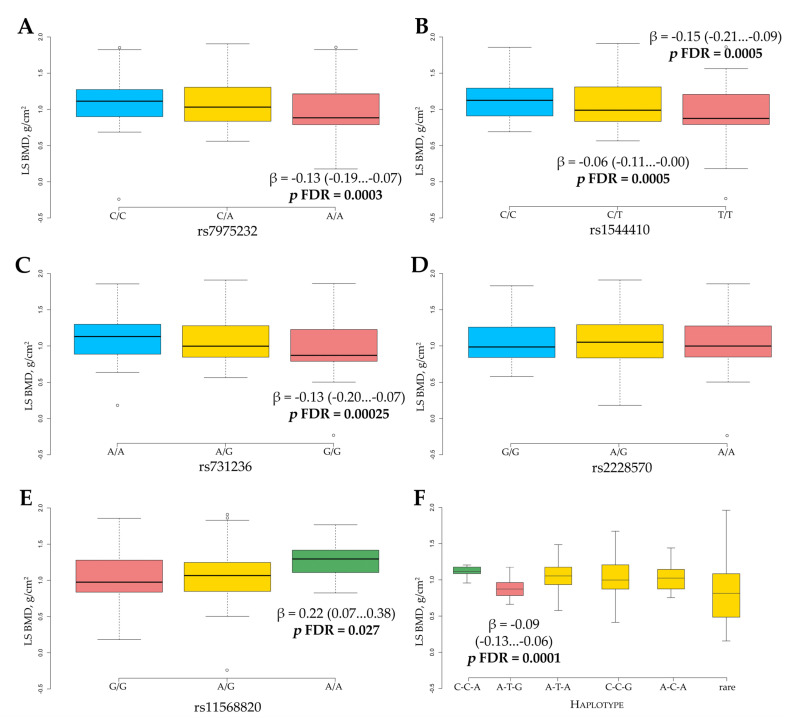
Lumbar spine (LS) BMD level in relation to *VDR* gene variants rs7975232 (**A**), rs1544410 (**B**), rs731236 (**C**), rs2228570 (**D**), rs11568820 (**E**), and rs7975232, rs1544410, and rs731236 haplotypes (**F**). For rs7975232, rs1544410, rs731236, and rs11568820 variants, gene/dose dependence was revealed. The rs11568820 was the only *VDR* gene variant with protective effect. *p*-values corrected for multiple testing using the FDR, *β* is the difference compared to the reference value. The data are presented as β (95% CI).

The association analysis between *VDR* gene single variants and haplotypes and LS BMD level was performed using linear regression on the combined cohort of study subjects (Figure 2).

The analysis revealed four *VDR* gene variants associated with L1-L4 BMD level. The observed difference in BMD level for rs7975232, rs1544410, and rs731236 minor homozygous genotypes compared to reference genotypes was almost the same (β = −0.13 g/cm^2^, *p* FDR = 0.0003; β = −0.15 g/cm^2^, *p* FDR = 0.0005, and β = −0.13 g/cm^2^, *p* FDR = 0.00025, respectively). Such a similar effect may be explained by the previously observed high LD between rs7975232, rs1544410, and rs731236. Interestingly, for all these variants, there was a gene/dose response revealed: the highest level of BMD was found for the bearers of major homozygous genotype, the intermediate level was found in heterozygotes, and the lowest level was found in minor homozygotes (Figure 2A–C). Quantitative analysis of the rs11568820 variant revealed a more significant association with LS BMD level (Figure 2E). The substitution of G to A was associated with much higher LS BMD values (β = 0.22, 95% CI 0.07‒0.38, *p* FDR = 0.027), suggesting that the rs11568820 A-allele has a protective effect. No significant association for rs2228570 was found with LS BMD (Figure 2D). As there was a strong positive LD between rs7975232, rs1544410, and rs731236 variants, we performed an analysis of association of their haplotypes with LS BMD level (Figure 2F). The bearers of the A-T-G haplotype showed a higher decrease in LS BMD compared to reference C-C-A (β = −0.09, 95% CI −0.13…−0.06, *p* FDR = 0.0001). No significant association with BMD was found for other haplotypes.

### 3.4. The Relationship between VDR Gene Variants and Serum 25(OH)D Level

**Figure 3:**
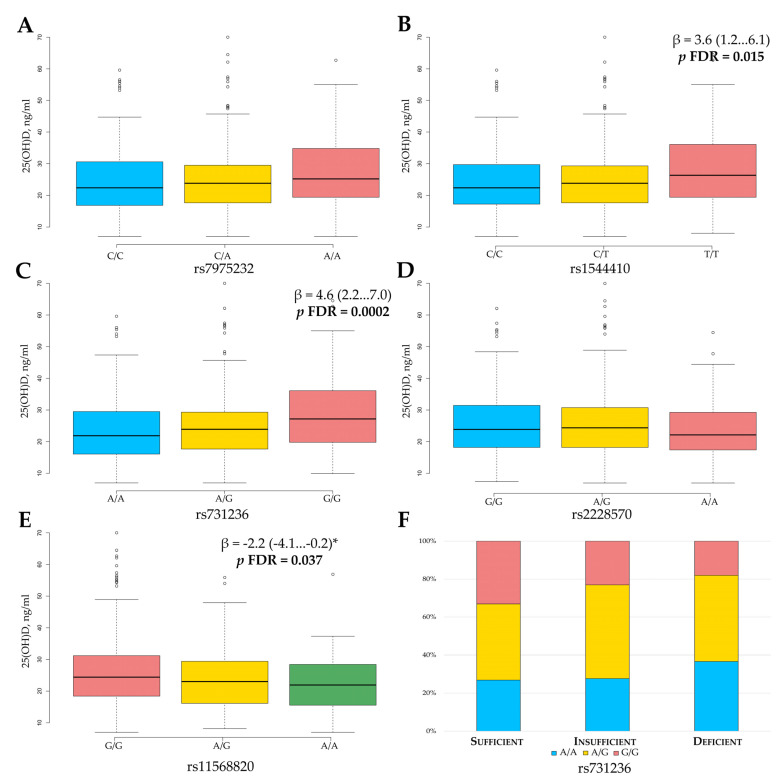
The association of serum 25-hydroxyvitamin D levels with *VDR* gene variants rs7975232 (**A**), rs1544410 (**B**), rs731236 (**C**), rs2228570 (**D**), rs11568820 (**E**), and rs731236 genotype distribution in groups based on Vitamin D status (**F**). For rs1544410, rs731236, and rs11568820 variants, gene/dose dependence was revealed. The rs11568820 was the only *VDR* gene variant with protective effect. *p*-values corrected for multiple testing using the FDR, *β* is the difference compared to the reference value. The data are presented as *β* (95% CI). * Dominant model of inheritance.

The effect of vitamin D is mediated through binding to a specific steroid receptor with the activity of a transcription factor, thus regulating the synthesis of protein actively participating in bone metabolism and maintaining calcium homeostasis. Variation in *VDR* gene may alter receptor functions, suggesting possible changes in serum 25(OH)D concentration. The relationship between *VDR* gene variants and serum 25(OH)D level is presented in Figure 3.

We revealed a statistically significant association of rs1544410, rs731236, and rs11568820 gene variants with the serum 25(OH)D level. The genetic effects of these three markers on baseline serum 25(OH)D level were gene/dose dependent. Interestingly, for rs1544410 (Figure 3B) and rs731236 (Figure 3C), the lowest 25(OH)D level was typical for the reference genotype, while it was intermediate for heterozygotes and the highest for the bearers of minor homozygous genotypes (ANOVA test *p* = 0.006 and *p* = 0.0005, respectively). For the bearers of the rs1544410 T/T genotype, the 25(OH)D concentration was 3.6 ng/mL higher compared to C/C genotypes (*p* FDR = 0.015), whereas for the bearers of the rs731236 G/G genotype, it was 4.6 ng/mL higher (*p* FDR = 0.0002). The opposite gene/dose relationship was revealed for the rs11568820 variant, when wild-type G/G homozygotes showed a higher 25(OH)D increase as compared to the A/G and A/A genotypes (Figure 3E).

We also assessed the distribution of each *VDR* variant of genotype in different groups of study participants according to vitamin D level (sufficient, insufficient, deficient). Using a two-tailed χ^2^ test, a statistically significant difference in genotype distribution between groups was revealed only for the rs731236 variant (χ^2^ = 12.8, *p* = 0.012, Figure 3F). The G/G genotype was over-represented in a group of participants with a “sufficient” state, while the A/A genotype was associated with vitamin D deficiency.

In order to increase the statistical power, we analyzed the association of serum 25(OH)D level with rs1544410, rs731236, and rs11568820 haplotypes (Table 4).

The haplotype analysis revealed five combinations (C-C-A, A-T-G, A-T-A, C-C-G, and A-C-A) with a frequency greater than 3%. The C-C-A haplotype was the most frequent (total frequency 44.8%) and was taken for reference. The total frequency of the A-T-G haplotype was 39.8%. For the bearers of the A-T-G haplotype, the 25(OH)D level was significantly higher compared to the reference haplotype (β = 2.0, 95% CI 0.7–3.4, *p* FDR = 0.017). No significant association with BMD level was found for other haplotypes.

## 4. Discussion

PMO is the most widespread type of disease, which causes serious medical, social, and economic difficulties for society. Research in the area of such complex (multifactorial) pathology aims to reveal both the environmental and genetic factors affecting disease development. According to numerous epidemiological studies, family and twin observations, up to 90% of OP cases are genetically determined [23]. The early detection of genetic factors, associated with predisposition to PMO, may help increase the prophylaxis and treatment effectiveness, although the evaluation of genetic background is complicated due to the involvement of multiple gene networks and their interaction with various environmental factors.

Vitamin D effects have been widely investigated in various populations with regard to its possible effect on PMO risk. The huge interest in vitamin D is explained primarily by its activity in calcium homeostasis, bone formation, and the regulation of bone mineral density. Vitamin D binds to a specific steroid receptor, which is coded by the *VDR* gene. VDR has a transcription factor activity and influences the expression activity of numerous target genes. The intensive study of the *VDR* gene made it possible to identify polymorphic variants that may lead to structural or functional changes in protein expression. Therefore, these variants may serve as potential clinical and diagnostic markers of bone muscular pathology. However, *VDR* gene studies continue and in a number of research studies, conflicting data on the distribution of genotype frequencies of the different loci of this gene were shown [12,13,23], thus providing a basis for further work in the area.

In the present study, we analyzed the association of the five most commonly studied *VDR* gene variants with PMO risk, BMD level, and serum 25(OH)D concentration in the Belarusian population. These SNVs were selected based on their previously established role in bone metabolism, modulation of *VDR* expression, and activity. Several previous research studies have suggested that *VDR* gene variants may influence the BMD level; this effect is variable and population-dependent [12,13], but complex studies on SNVs association with vitamin D level are still lacking.

The observed differences in body weight and BMI between groups are quite expected, as decreased BMI and body weight are well-known risk factors of OP. The revealed average level of BMI in the control group can be explained by the population features of the Belarusian cohort.

We compared the allele distribution of analyzed *VDR* gene variants with that of the populations included in the gnomAD database [22]. For rs7975232, rs1544410, rs731236, and rs11568820, our study revealed no significant difference in minor allele frequency (MAF) as compared to European cohort. However, MAF (allele A) for rs2228570 was significantly higher (48%) as compared to the gnomAD European cohort (37%). This difference may be due to the cohort size, ethnicity, or gender specificity.

The analysis of our data revealed a strong association of rs7975232, rs1544410, and rs731236 variants with PMO risk, where A/A, T/T, and G/G genotypes, correspondingly, were over-represented in the patients group. This association remained even after correction for cofounding factors, suggesting the predominant contribution of hereditary factors and their role as potential markers of PMO risk. For all these three *VDR* gene variants located in the ligand-binding domain at the 3′-end of the gene, a strong linkage disequilibrium was found. The located upstream rs2228570 in the DNA-binding domain and rs11568820 in the promoter region did not show LD. These data are in agreement with previous studies on European populations [24,25]. Despite the fact that a strong LD was revealed between rs7975232, rs1544410, and rs731236 markers, this linkage is not full, and it is not possible to predict the alleles of one SNV, knowing the alleles of other SNVs. Perhaps, this explains that the risk of PMO in haplotype analysis (for the bearers of A-T-G haplotype) was lower compared to single gene analysis (for the bearers of rs7975232 A/A, rs1544410 T/T, and rs731236 G/G genotypes).

When examined with respect to the interaction with LS BMD, in addition to rs7975232, rs1544410, and rs731236, a statistically significant association was also revealed for rs11568820. However, there was no effect revealed for rs2228570. Interestingly, a dose effect was observed for all these variants, which is very important. Women with the rs7975232, rs1544410, and rs731236 minor homozygous genotypes had the lowest BMD level compared to bearers of other genotypes. In contrast, the rs11568820 A/A genotype was associated with increased BMD level, suggesting its protective effect. Unlike other gene variants, rs11568820 is located in the promoter region of the *VDR* gene, and previously, it was reported that the A-allele relates to higher promoter activity in vitro [26]. The same effect on BMD was observed in Slovenian women with PMO [27]. Previously, various studies also reported a significant association with LS BMD of rs7975232 [28] and rs1544410 [27] in Caucasian women. The most unexpected result of our study was the absence of a statistically significant association of rs2228570 with BMD level, although it was previously reported in Caucasian women with PMO [28]. On the other hand, our results are consistent with a huge meta-analysis, which was performed recently [12]. Such differences within the same populations may be explained by the involved environmental factors, gene–gene and gene–environment interactions or sample sizes. The rs2228570 variant plays an important role in message stability and post-transcriptional processes in the *VDR* gene, and due to its functions, more extensive research is required. Interestingly, our results showed consistency: rs7975232, rs1544410, and rs731236 were associated both with a decreased risk of PMO and high levels of LS BMD. This result may be explained by strong LD between variants. Moreover, the simultaneous increase of PMO risk and decrease in the lumbar BMD level for the bearers of the A-T-G haplotype was observed. The same association was previously revealed in Polish [28] and Dutch [29] populations. High LD creates additional difficulties, since the exact gene variant with a causative effect is unknown.

Possibly, the most interesting part of the study is the analysis of the association of *VDR* gene variants with vitamin D status. Such an interest in vitamin D is explained by importance for bone health and by its pleiotropic biological action, which mediates predisposition not only to bone muscular diseases but also to many other complex diseases. Recent studies have suggested that SNVs within the *VDR* gene may influence the level or activity of vitamin D. In the present study, we revealed a statistically significant gene/dose association of rs1544410, rs731236, and rs11568820 variants with plasma 25(OH)D level. The rs1544410 T/T and rs731236 G/G genotypes are associated with an increased level of plasma vitamin D compared to the reference genotype, while rs11568820 combined A/G+A/A genotype bearers were characterized by a decrease. The same association was previously revealed in Caucasian [30,31], Chinese [32], and Egyptian [33] populations. In the analysis of *VDR* gene variants distribution within groups according to vitamin D status (sufficient, insufficient, deficient), only rs731236 revealed a significant association, whereas the bearers of A/A genotypes were over-represented in the group of patients with deficiency. These data are confirmed by a recent study on response to vitamin D supplementation [15]. In haplotype analysis, which was performed to test whether combinations of different *VDR* gene variants may predict serum 25(OH)D concentration, the highest level of serum vitamin was found in bearers of A-T-G alleles, which are associated with PMO risk and low LS BMD. Located at the 3′-end of the *VDR* gene, rs7975232, rs1544410, and rs731236 variants are associated with the different-length polyadenylate sequences and affect the stability of mRNA, while the rs11568820 variant could change the transcription activity of the promoter region of the gene [34]. Thus, we may hypothesize that the increased level of circulating 25(OH)D in patients compared to controls (Table 1) may be explained by the fact that unfavorable *VDR* genotypes were over-represented in the PMO group, possibly altering metabolic feedback loops or the effectiveness of vitamin metabolism. This hypothesis is confirmed by the fact that the level of VDR mRNA was remarkably reduced in bearers of the rs1544410 T/T genotype compared to individuals with the C/C genotype [35]. The bearers of favorable rs7975232 C-, rs1544410 C-, and rs731236 A-alleles have higher VDR receptor expression, thus leading to increased vitamin D metabolism. The possible mechanism may include an alteration of vitamin D-mediated gene expression by differential activity of the VDR receptor. Specific SNVs may decrease the activity of a wide range of enzymes involved in the production and elimination of 25(OH)D, promoting an increase in circulating serum 25(OH)D level and a simultaneous decrease in intercellular 1,25-dihydroxyvitamin D concentration, resulting in adverse health effects caused by vitamin D deficiency despite higher circulating 25(OH)D concentration [14]. This hypothesis requires further confirmation in other studies.

The remaining rs2228570 with a previously established role in vitamin D-related pathways revealed no association with plasma 25(OH)D level in our cohort. This variant, which is located in the second exon, forms a second methionine start site, producing a shorter protein receptor, which displayed higher transcriptional activity. Our results are contrary to studies where in bearers of the rs2228570 G/G genotype, an increase in 25(OH)D concentration [36] and affected 25(OH)D hydroxylation [37] was reported. Although we found no significant association for this variant with PMO risk, LS BMD, and 25(OH)D level in the present study, broader research with a bigger cohort may confirm this variant as a marker for personalized medicine purposes.

Nevertheless, a very interesting result is that the variants associated with low LS BMD levels were also associated with high 25(OH)D levels and vice versa. Such a tendency is found for all analyzed SNVs, suggesting the differential action of vitamin D on the local cell level in bones and circulating in plasma. At least rs7975232, rs1544410, rs731236, and rs11568820 might help to identify individuals with increased PMO risk and vitamin D status. The revealed considerable variation in serum 25(OH)D in individuals with different *VDR* genotypes further suggest that a one-size-fits-all approach to vitamin D supplementation may not be appropriate. For more accurate evaluation of the association of *VDR* gene polymorphism with predisposition to PMO, the analysis of various environmental factors, such as diet, sun exposure, exercise, and other is required. The relationship between gene variation and vitamin D status also requires further investigation. In addition, other gene variants within vitamin D pathway, as well as epigenetic factors can also play a significant role in the pathogenesis of disease.

## 5. Conclusions

We investigated the association of five major *VDR* gene variants with PMO risk, BMD level, and serum 25(OH)D concentration. Our study shows novel data on vitamin D genetics and homeostasis, particularly on the significant association of four markers with BMD and 25(OH)D levels. *VDR* rs7975232, rs1544410, rs731236, and rs11568820 might be taken into consideration for individual PMO risk assessment and the development of personalized recommendations for the optimization of vitamin D supplementation.

## Figures and Tables

**Table 1:** Clinical characteristics of study subjects.

Clinical Characteristic	PMO Patients	Control	*p*-Value
n (%)	355 (59.0)	247 (41.0)	
Age, years	63.0 (57.0; 70.0)	62.0 (58.0; 67.0)	0.12
Age at menopause, years	50.0 (47.0; 52.0)	50.0 (48.0; 52.0)	0.32
Weight, kg	66.0 (58.0; 74.0)	81.0 (73.0; 93.0)	**0.0001**
Height, cm	160.0 (156.0; 165.0)	159.0 (155.0; 164.0)	0.20
BMI, kg/m^2^	25.5 (22.5; 28.7)	31.6 (28.3; 36.2)	**0.0001**
LS BMD, g/cm^2^	0.9 (0.8; 0.9)	1.3 (1.2; 1.4)	**0.0001**
LS T-score	−2.6 (‒3.2; −2.0)	0.7 (−0.1; 1.3)	**0.0001**
LS Z-score	−1.1 (‒1.7; −0.5)	1.3 (0.6; 2.1)	**0.0001**
FN BMD, g/cm^2^	0.8 (0.7; 0.9)	1.1 (1.0; 1.2)	**0.0001**
FN T-score	−1.7 (−2.4; −1.1)	0.4 (−0.1; 1.0)	**0.0001**
FN Z-score	−0.7 (−1.2; −0.1)	1.0 (0.4; 1.6)	**0.0001**
25-hydroxyvitamin D (25(OH)D), ng/mL	27.0 (21.1; 35.8)	19.9 (15.2; 26.4)	**0.0001**
Fractures in history	49 (13.8%)	7 (2.8%)	

The data are presented as median (25%; 75% interquartile range). BMI, body mass index; LS, lumbar spine; FN, femur neck; BMD, bone mineral density; PMO, postmenopausal osteoporosis. The values highlighted in bold indicate a significant association.

**Table 2:** The Hardy–Weinberg equilibrium *p*-values and distribution of genotype frequencies of *VDR* gene variants in patients with postmenopausal osteoporosis and control.

Gene Variant	Genotype	PMO *n* = 355		CON *n* = 247		OR (95% CI)	Adjusted OR (95% CI) ^1^
		%	HWE	%	HWE		
rs7975232	C/C	23.7	0.17	31.2	0.7	Ref.	Ref.
	C/A	45.9		48.2		1.3 (0.9–1.9)	1.3 (0.8–2.1)
	A/A	30.4		20.6		1.9 (1.2–3.1) *	2.1 (1.3–3.6) *
rs1544410	C/C	25.4	0.24	36.4	0.6	Ref.	Ref.
	C/T	46.7		46.6		1.5 (0.9–2.1)	1.6 (1.0–2.4) *
	T/T	27.9		17.0		2.4 (1.5–3.8) **	2.1 (1.2–3.6) *
rs731236	A/A	24.2	0.4	37.2	0.51	Ref.	Ref.
	A/G	47.6		45.8		1.6 (1.1–2.3) **	2.1 (1.3–3.3) **
	G/G	28.2		17.0		2.6 (1.6–4.1) **	2.3 (1.4–4.0) **
rs2228570	G/G	27.9	0.17	26.7	1.0	Ref.	Ref.
	A/G	46.2		50.2		0.9 (0.6–1.3)	0.9 (0.6–1.5)
	A/A	25.9		23.1		1.1 (0.7–1.7)	1.0 (0.6–1.8)
rs11568820	C/C	69.0	0.02	66.4	0.68	Ref.	Ref.
	C/T	30.1		29.6		0.9 (0.6–1.3)	1.2 (0.8–1.8)
	T/T	0.9		4.0		0.2 (0.1–0.7)	0.2 (0.1–1.0)
	C/T+T/T ^2^	31.0		33.6		0.8 (0.6–1.3)	1.1 (0.7–1.6)

PMO, postmenopausal osteoporosis; CON, control; HWE, Hardy–Weinberg equilibrium; OR, odds ratio; CI, confidence interval; Ref., referent value; * *p* FDR < 0.05, ** *p* FDR < 0.01; ^1^ Adjusted by confounding factors (BMI); ^2^ Dominant model of inheritance used due to low minor allele frequency. The values highlighted in bold indicate a significant association.

**Table 3:** Haplotype analysis of *VDR* rs7975232, rs1544410, and rs731236 gene variants in patients with postmenopausal osteoporosis (PMO) and control (CON) group.

Haplotype	Frequency, %		Haplotype Score	*p* FDR	Logistic Regression	
	PMO	CON			OR (95% CI)	*p*
C-C-A	38.6	49.6	**−3.53**	**0.001**	Ref.	-
A-T-G	44.9	31.7	**4.41**	**0.00005**	**1.8 (1.4–2.3)**	**0.0001**
A-T-A	4.0	7.1	−1.78	0.12	0.9 (0.6–1.4)	0.63
C-C-G	5.7	4.1	0.84	0.50	1.6 (0.9–2.7)	0.11
A-C-A	4.2	3.5	0.15	0.88	1.3 (0.8–2.2)	0.34
Rare *	2.6	4.0	-	-	-	-

OR, odds ratio; Ref., referent value; CI, confidence interval; FDR, false discovery rate. * Rare haplotypes—other possible haplotypes with total frequency less than 3%. The values highlighted in bold indicate a significant association.

**Table 4:** The association of *VDR* rs7975232, rs1544410, and rs731236 haplotypes with serum 25(OH)D levels.

Haplotype	Frequency, %	25(OH)D, ng/mL (mean ± SE)	Linear Regression	
			β (95% CI)	*p* FDR
C-C-A	44.8	21.7 ± 0.7	Ref.	-
A-T-G	39.8	23.7 ± 0.8	**2.0 (0.7…3.4)**	**0.017**
A-T-A	4.4	25.4 ± 1.4	−1.7 (−4.4…1.0)	0.35
C-C-G	4.2	23.5 ± 1.5	0.2 (−2.8…3.1)	0.91
A-C-A	3.6	24.0 ± 1.5	−0.3 (−3.2…2.6)	1.0
Rare *	3.2	27.9 ± 4.3	4.2 (1.1…7.4)	0.02

Ref., referent value; SE, standard error; β, difference compared to reference value; CI, confidence interval. * Rare haplotypes—other possible haplotypes with total frequency less than 3%. The values highlighted in bold indicate a significant association.

## Data Availability

The data supporting the conclusions of this manuscript will be made available by the authors to any qualified researcher.
